# Reviewed article: Biase, TMMA; Silva, MT; Galvão, TF. Selective reporting of adverse renal reactions associated with selective cyclooxygenase-2 inhibitors in randomized clinical trials: a cross-sectional study, 2023. Epidemiol Serv Saude. 2026:35;e20260191

**DOI:** 10.1590/S2237-96222026v35e20260191.c

**Published:** 2026-11-09

**Authors:** Nathalia Sernizon Guimarães

**Affiliations:** 1 Universidade Federal de Minas Gerais, Belo Horizonte, MG, Brazil Universidade Federal de Minas Gerais Belo Horizonte MG Brazil

## First round comments

Diante os cinco pareceres recebidos recomendamos que o artigo passe por ajustes pontuais, conforme detalhado a seguir.

Do ponto de vista conceitual e metodológico, recomenda-se aprimorar a definição operacional do desfecho “relato seletivo”, esclarecendo de forma mais robusta como essa classificação foi realizada e se houve comparação sistemática com protocolos, registros de ensaios ou planos de análise. Sem esse detalhamento, há risco de erro de classificação e dificuldade em distinguir relato seletivo de relato incompleto. Ainda nos métodos, é necessário explicitar claramente a dicotomização da variável dependente logo no início da seção.

A análise estatística também deve ser revista ou, ao menos, melhor discutida, considerando que se limita a comparações bivariadas sem controle para potenciais fatores de confusão. Caso análises multivariadas não sejam viáveis, essa limitação deve ser explicitada. Além disso, o uso do tempo entre submissão e aceite como proxy de qualidade da revisão por pares é questionável; sugere-se adotar a denominação “tempo de processamento editorial” e discutir suas limitações, inclusive quanto à influência de fatores externos não controláveis. De modo semelhante, o uso do fator de impacto de 2022 para artigos publicados entre 1999 e 2018 deve ser problematizado quanto à validade interpretativa.

Na introdução, sugere-se maior precisão conceitual e ajustes de redação, incluindo: revisão de termos como “tradicionais”, melhor caracterização da gravidade dos desfechos (por exemplo, substituindo “riscos desnecessários” por expressão mais forte), inclusão explícita do conceito de relato seletivo e aprofundamento da contextualização histórica, especialmente no caso da retirada do rofecoxibe, incluindo aspectos relacionados à omissão de eventos adversos em estudos clínicos.

Na seção de resultados, recomenda-se incluir uma descrição mais detalhada do desfecho (relato seletivo), preferencialmente em tabela ou material suplementar. Já na discussão, é necessário ampliar a interpretação dos achados, contemplando todos os resultados estatisticamente significativos - como diferenças relacionadas à duração dos estudos e à condição clínica (por exemplo, osteoartrite) - e evitar extrapolações que não sejam plenamente sustentadas pelos dados, especialmente no que se refere à influência de políticas editoriais e patrocínio. Sugere-se também reorganizar trechos da discussão, incorporando melhor as limitações do estudo.

O resumo e o título precisam ser ajustados às normas da revista: o resumo deve ser estruturado (Objetivo, Métodos, Resultados e Conclusão) em parágrafo único, e o título deve incluir tema, delineamento, local e período. Ainda no resumo, recomenda-se maior precisão na redação dos objetivos e na descrição dos resultados.

Há também diversas adequações formais e editoriais a serem realizadas, incluindo:

revisão da lista de referências (limite de 30);

padronização das citações no formato Vancouver com colchetes;

ajustes nas tabelas (títulos autossuficientes, organização de cabeçalhos, alinhamento, remoção de notas desnecessárias, padronização de termos e formatação);

explicitação de siglas e termos;

revisão de descritores conforme DeCS;

correção de espaçamentos e sinais;

adequação do uso de termos estrangeiros;

inclusão adequada da seção de disponibilidade de dados com citação formal;

revisão de similaridade textual para garantir originalidade conforme exigências da revista.

Recommendation: Minor revision 

